# A mixed method study on Chinese primary school EFL teachers’ preparation, affecting factors and support needed to implement intercultural foreign language teaching

**DOI:** 10.1371/journal.pone.0284146

**Published:** 2023-04-07

**Authors:** Huang Wang, Tianyuan Xu, Mengxue Zhang

**Affiliations:** 1 School of Foreign Studies, Hunan First Normal University, Changsha, China; 2 School of Foreign Languages, Zhongnan University of Economics and Law, Wuhan, China; Tallinn University: Tallinna Ulikool, ESTONIA

## Abstract

Cultivating intercultural competence is a long-term and staged process requiring the efforts of all counterparts in the education field from primary school up to university. Currently, most research on intercultural education in China focuses on the tertiary education context, and little attention has been paid to elementary education as well as primary school EFL teachers. Against this background, this study intends to investigate Chinese primary school EFL teachers’ preparedness for intercultural foreign language teaching (IFLT), its influencing factors, and the support teachers need to implement IFLT. A convergent mixed method was used in this study. Data was collected through questionnaires and interviews, SPSS and the thematic analysis method were used to analyze the data. Via both quantitative and qualitative methods, this empirical study found that: 1. Primary school EFL teachers are not well prepared for IFLT; 2. Textbooks, the current evaluation system, teachers’ lack of literacy in intercultural competence, insufficient teacher training on intercultural competence, and teachers’ lack of time and energy are five major factors that constrain the implementation of IFLT; 3. Support from school administration, the construction of intercultural-related materials and resources, and practice-oriented teacher training are the three main support teachers need. Based on these findings, the role of textbooks, experience abroad and general materials on culture in promoting IFLT were discussed. At last, implications and future research directions were proposed.

## Introduction

With deepening development in the 21st century, opportunities and challenges coexist against the backdrop of globalization. Gaining and mastering intercultural knowledge and skill have been widely regarded as one of the educational goals in many countries, and so in China. In the field of foreign language teaching, *Guidelines for College English Teaching*, N*ational Standards of Teaching Quality for Undergraduate English Major*s and *English Curriculum Standards for Compulsory Education* (2022 version) (hereinafter referred to as Curriculum Standards), all those national documents have emphasized the importance and urgency of developing students’ intercultural competence from the perspective of policy orientation and national education decision-making. The *Curriculum Standards*–the programmatic document for English courses at the elementary education stage, lists ‘cultural awareness’ as one of the key competencies of English subjects and defines it as ‘the intercultural cognition, attitude and behavioral choices of students in the new era’. It can be seen that cultivating pupils’ intercultural competence in elementary education has become one of the teaching requirements at the national level.

Although China has become the second largest economy in the world, it is still a developing country in terms of GDP per capital. This is especially true for the central and western parts of China. Short-term foreign exchange, travel, and study abroad and other methods that have been proven effective in many countries to cultivate students’ intercultural competence cannot be achieved in those area due to their limited resources. That is to say, in the Chinese context, the major field to cultivate students’ intercultural competence lies in schools and teachers, especially foreign language teachers. Hence, their understanding of intercultural foreign language teaching (IFLT) is very important for the realization of cultivating students’ intercultural competence in China.

The cultivation of intercultural competence is a long-term process, and it can not be achieved overnight. Taking into the long-term and staged nature of its cultivation, China has put forward the goal of cultivating pupils’ intercultural competence in elementary education. It is against this background that this study put its focuses on primary school EFL teachers.

This study investigated how well-prepared Chinese primary school EFL teachers are to implement IFLT, its affecting factors and what we can do to help teachers better achieve this challenging goal. Unlike other similar studies in investigating teachers’ beliefs and practices through questionnaires alone, the current research values teachers’ voices in answering the above questions. By presenting front-line teachers’ answers to the above questions via both quantitative and qualitative data, we can better identify the gap between ‘ideal’ and ‘reality’, and better position the ‘problem’—how can a good policy change be implemented, rather than being stuck at the ‘last mile’.

## Literature review

### Theories and models of intercultural competence

Intercultural competence is the fundamental concept in intercultural communication studies [1]. Scholars worldwide have been constructing models or building theories of intercultural competence from various perspectives. Intercultural competence has been conceptualized differently by many scholars depending on their theoretical backgrounds or study subjects [2]. Spitzberg and Changnon conducted a systematic review of intercultural competence theories and models in the existing literature. They divided the current models of intercultural competence into five categories: compositional, co-orientational, developmental, adaptational, and causal process [3]. From such a classification, we could glimpse the complexity and diversity of intercultural competence concepts.

Deardorff’s study documented outstanding intercultural experts’ consensus using the Delphi methods. Among all the definitions provided, the top-rated definition of intercultural competence was ‘the ability to communicate effectively and appropriately in intercultural situations based on one’s intercultural knowledge, skills, and attitudes’ [4]. Deardorff also found that the definition of intercultural competence that 24 U.S. international institutional administrators found most acceptable was the one drawn from Byram’s study. In Byram’s model, intercultural communicative competence is composed of linguistic competence, sociolinguistic competence, discourse competence, and intercultural competence, which further includes attitudes, knowledge, skills of interpreting/relating, skill sf discovering/interaction, and critical cultural awareness [5]. Byram’s model proposes attainable educational objectives in foreign language education, and aims to produce intercultural speakers instead of native-like speakers. At the same time, it is also a model highlighting the importance of foreign language proficiency. The two characteristics made Byram’s model one of the most highly cited, widely tested, and practiced models in educational and foreign language teaching contexts [4]. Therefore, it is used as the theoretical framework of the current study.

### English curriculum reform in China over the past four decades

Since implementing the Reform and opening-up policy in 1978, China’s global connectivity has constantly risen. In 1982, the Chinese Ministry of Education formally proposed English as the first foreign language in school education, and English education in China was then put on a fast track. In the college entrance examination, which is considered the baton of Chinese education, there are six foreign languages (English, Russian, Japanese, Germany, French, and Spanish) that can be chosen. According to the data from the Ministry of Education, in 2022, 19.13 million candidates participated in the college entrance examination. However, only 300,000 chose the other five foreign languages as exam subjects, showing English’s dominant position as the first foreign language in Chinese educational context.

English has been a necessary part of school curricula at various educational levels for the past forty years. Therefore, ongoing reforms of the English curriculum have been made to keep up with the requirements raised by the new era [6]. Li summarized the historical change in the objectives and characteristics of English language teaching in China from 1978–2012 [7]. During 1978–1992, the objectives of English teaching emphasized cultivating students’ reading ability and mastering English language knowledge. Between 1993 and 2000, attention began to be paid to developing students’ overall competence, and language learning began to include content beyond the structure of language knowledge. In 2000, developing students’ understanding of cultural differences and their awareness of the world through English education was proposed for the first time. From 2000 to 2012, the most notable sign of English curriculum reform in China was the replacement of the syllabus with curriculum standards. The curriculum standards set requirements not only for knowledge, competence and skills, but also for emotions, attitudes, values and intercultural communicative awareness. More importantly, the curriculum standards placed these non-intellectual requirements on an equally important footing with those intellectual ones. At this stage, the objectives of the English language curriculum break away from the traditional instrumentalist ideology of knowledge orientation and highlight the close relationship between language and culture, requiring teachers to properly deal with the relationship between the two in their teaching process. As stated in the latest documents of the *National English Curriculum Standards* (2011 version and 2022 version), English is now taught as a subject from grade three in primary schools throughout China. The *Curriculum Standards* sets goals for English education from the language proficiency, learning strategy, thinking quality, and cultural awareness. The *Curriculum Standards* states that primary school students are expected to reach Level 2 after three or four years of English study from grades 3 to 6. According to the 2022 *Curriculum Standards*, cultural awareness in the initial stage is to help students understand the outstanding civilizational achievements of different countries and the similarities and differences between Chinese and other cultures.

Reviewing the two most current *Curriculum Standards* allows us to conclude that the equal emphasis on the instrumental and humanistic nature of English is becoming increasingly important in English teaching, as opposed to the conventional instrumental function. Developing students’ intercultural competence has become one of the key objectives of English language teaching in China, starting from the elementary education stage. Such emphasis aims to meet the demands of students’ personal development to prepare them for the challenges of living in a diverse and dynamic multicultural environment.

### Intercultural Foreign Language Teaching (IFLT)

Although cultivating learners’ intercultural competence has become a major goal for foreign language education, cultural teaching has always been a weak link in English education, especially at the compulsory level. English teaching for pupils has remained strongly instrumental, almost obscuring the language’s cultural meaning and humanistic spirit [8]. As observed in many studies, culture is simply regarded as the encyclopedic knowledge to be taught, and students need to memorize that knowledge rather than explore it by themselves [9]. Liddicoat thinks that intercultural language pedagogy includes two aspects. The first is that it engages with the interrelatedness of language culture and learning and the different languages and cultures in the classroom. The second aspect is recognizing that there are always at least two languages in use at every moment—the target language and the learners’ first language [10]. However, due to the profound influence of Communicative Language Teaching (CLT), most English teachers are pushing for an all-English classroom. They attempt to exclude the learners’ mother tongue as much as possible because it is seen as a barrier to acquiring a foreign language. Scholars from home and abroad compare intercultural foreign language teaching and traditional, knowledge-oriented teaching methods [11–13]. The difference between the two methods is that while traditional language teaching emphasizes the acquisition of linguistic knowledge; IFLT pays attention not only to language proficiency but also to the home culture, foreign cultures, and culture in general. In short, IFLT aims to integrate foreign language teaching and cultural teaching to promote the joint development of students’ language ability and intercultural competence [13]. During the process, language teachers are no longer concerned only with skills or knowledge which appear to be value-free; they should provide learners with a safe learning environment where students are willing to take risks to critically reflect on their own culture and the culture in question [14]. Such a pedagogical change obviously requires a change in the role and teaching method of teachers and therefore places new demands on foreign language teachers.

Furthermore, although researchers’ interest in intercultural foreign language teaching has been on the rise, research on IFLT in elementary education is insufficient. Compared with abundant studies in tertiary education, the cultivation of young learners’ intercultural competence is under-researched [13, 15]. In contrast to the lack of research on IFLT at the primary level, numerous studies have confirmed the importance and necessity of intercultural education for children. Children aged 3–4 years already begin categorizing people according to race and ethnicity, and obvious comparisons between ethnic groups emerge [16, 17]. Powlishta *et al*. [18] emphasize the need for intercultural education for children, as stereotypes are formed in childhood and cemented in the adolescent stage. The development of intercultural competence not only enhances children’s sense of identity but also stimulates their sensitivity to other cultures and their awareness of openness and tolerance [19, 20]. In addition, a meta-analysis found a significant decline in the strength of intervention effects on students’ intercultural competence from K-12 to tertiary education, which again proved the importance and necessity of implementing IFLT at the elementary level [21].

### Intercultural foreign language teachers

To achieve the transformation from traditional language teaching to intercultural foreign language teaching, the role of language teachers as agents of change are essential. Hence, efforts to understand their position and opinions are important as foreign language teaching is moving towards more comprehensive goals. Intercultural competence is not something that learners can ‘pick up by themselves when they go to a foreign country’ [10]. It requires the efforts of foreign language teachers to equip their students with the set of attitudes, knowledge, and skills needed in intercultural communication. In this way, it implies that language teachers should become intercultural competent themselves and be prepared for the new challenges raised by the new era [12, 22].

Following this line of thought, teachers’ beliefs and attitudes toward IFLT have received much attention. Representative research abroad includes Sercu and Bandura’s survey of the cultural teaching beliefs and attitudes of 424 secondary school foreign language teachers [23]; Oranje & Smith’s survey of the intercultural language teaching beliefs of language teachers in 76 high schools in New Zealand [24]. Chinese representative research includes Shao’s survey on the intercultural sensitivity and cultural teaching status of 35 English teachers in 4 high schools in Jiangsu Province [25]. Zhang’s study on the beliefs of intercultural competence of foreign language teachers in universities in Wuhan [26] and Han’s research of intercultural communicative competence and intercultural competence teaching cognition of 1081 English teachers in 39 universities across China [27].

Those studies mentioned above have found that foreign language teachers’ beliefs at home and abroad have not changed substantially. Although teachers mostly agree on the critical role of culture or intercultural competence in foreign languages, they have uncertain attitudes and ambivalent emotions about integrating cultural dimensions into language teaching. The dominant teaching method is teacher-centered, and the primary purpose is to impart the language and cultural knowledge of the target country. Language competence is still the top priority, while intercultural competence is an ‘add-on’ part of language education.

In addition, studies also found that teachers have little acquaintance with culture theory and need more pedagogical training in cultural teaching [28]. Their approaches to culture are mainly essentialist and nationalist, based on Communicative Language Teaching method [29]. Due to the lack of awareness and knowledge of intercultural education, teachers attach importance of the English language to native speakers’ language and cultural norms [30], and they tend to link cultural content directly to English-speaking countries [31].

The results of the studies mentioned above indicate that although the cultivation of learners’ intercultural competence has become an educational objective at the national level, foreign language teachers do not keep up with the trend to some extent.

### Factors that may affect teachers’ implementation of IFLT

Studies have shown that a range of personal, sociocultural, and institutional variables, including a person’s cultural background, work experiences, and school community, affect teachers’ implementation of teaching intercultural competence [32, 33]. For example, studies found that teachers’ intercultural encounters significantly impacted their beliefs on intercultural education [32, 34], and negative inter-group contact was associated with reduced intercultural competence [35–37]. Another study discovered that teachers’ problematic understanding of culture limited teachers’ identification of intercultural objectives in language education [38]. A study also found that teachers did not understand the concept of intercultural competence [39], and they lumped members of the target culture together [31]. Research by Maijala identified three significant challenges for cultural teaching: how to incorporate culture into lessons, using the target language, and using personal cultural experiences in a pedagogically helpful manner [40].

Contextual factors were also discovered to have greatly affected language teachers’ instruction of intercultural competence compared to the individual factors listed above. Several studies proved that the lack of time available to teach intercultural content, appropriate teaching materials, and teacher training in teaching intercultural competence could hinder EFL teachers’ willingness to implement IFLT [41–43]. Salli’s study proved the effectiveness of a training session in achieving the ICC goals in foreign language education through action research [44]. While Safa and Tofighi’s study implied that teacher training programs in Iran mediate the development of intercultural competence theory aspects, they do not result in the development of the necessary skills for the implementation of intercultural competence in pedagogical practices [45]. The current teacher training programs in the field of interculturality encourage participants’ reflection, but it has the weakness of being non-contextual, inflexible and dominated by theoretical learning [46]. Through the analysis of research papers, Romijn *et al*. argued that the effectiveness of an embedded and contextual approach in which reflection is guided and enactment is fostered is most likely to enhance teachers’ intercultural competence [47]. Since textbooks are the primary tools teachers use to teach foreign languages, another essential facet of IFLT research is the teaching materials. Many studies examined the intercultural content in textbooks used in educational contexts [48–50]. Song’s study investigated the strength and weaknesses of the cultural content in one of the most extensively used textbooks in China. She found that the strength of this textbook lies in its great deal of general cultural content, but it severely neglected the source culture compared to the target culture [49]. Minh and Phuong discovered that textbooks in Vietnam do not provide students with opportunities for intercultural learning, and the books also tend to present a static view of culture [51]. Teachers also felt the scattered, overgeneralized, and stereotypical culture presentation in textbooks [52]. In addition, several studies have pointed out the importance of involving more outer and expanding circle countries in textbooks [53] as well as general cultural knowledge [15]. Although a study demonstrates that teachers can successfully use EFL textbooks along with possible teaching resources that include cultural values to foster learners’ intercultural competence [54], most research indicates that the currently used textbooks are not appropriate for the cultivation of intercultural competence [51, 55].

### Gap in the literature review

From the above literature review, we can find that the state has emphasized the importance of developing students’ intercultural competence through foreign language education from its policy orientation. The Curriculum Standards also set out the levels of intercultural competence that students are expected to achieve by the end of primary school. However, this is in contrast to the lack of research on IFLT at primary level. In addition, foreign language teachers’ role is of essential importance in the implementation of such a transformation. However, most studies on foreign language teachers mentioned above put their research contexts in secondary or higher education, and none of them has been focused on primary school EFL teachers specifically. There is not enough research on the IFLT at primary school levels and among primary school EFL teachers. The lack of research in this area does not match its importance. Furthermore, most of the related studies use the questionnaire method as the main way to collect data. The questionnaire allows for a large amount of data to be obtained about the current state of teachers’ opinions of IFLT, but it is limited in that it does not explain why teachers think this way. Another limitation of the questionnaire is that teachers’ answers are limited by the options provided in the questionnaire, making it difficult to get insightful answers from teachers. In other words, what do front-line language teachers consider to be the factors that facilitate or hinder IFLT? What support do front-line teachers need in order to get the national policy implemented? Based on the above discussion, this research targets primary school EFL teachers in China, trying to answer the following research questions using convergent mixed methods:

1. How prepared are Chinese primary school EFL teachers to meet the requirement of IFLT raised in the *Curriculum Standards*?
2. What affects Chinese primary school EFL teachers’ implementation of IFLT?
3. What support do Chinese primary school EFL teachers need to implement IFLT?

## Methodology

### Research design

A mixed methods design was used in the current study. A mixed methods design is one that ‘include at least one quantitative method (designed to collect numbers) and one qualitative method (designed to collect words)’ [56]. The researcher combined qualitative and quantitative approaches in order to have a ‘breadth and depth understanding and corroboration’ [57].

**Fig 1:**
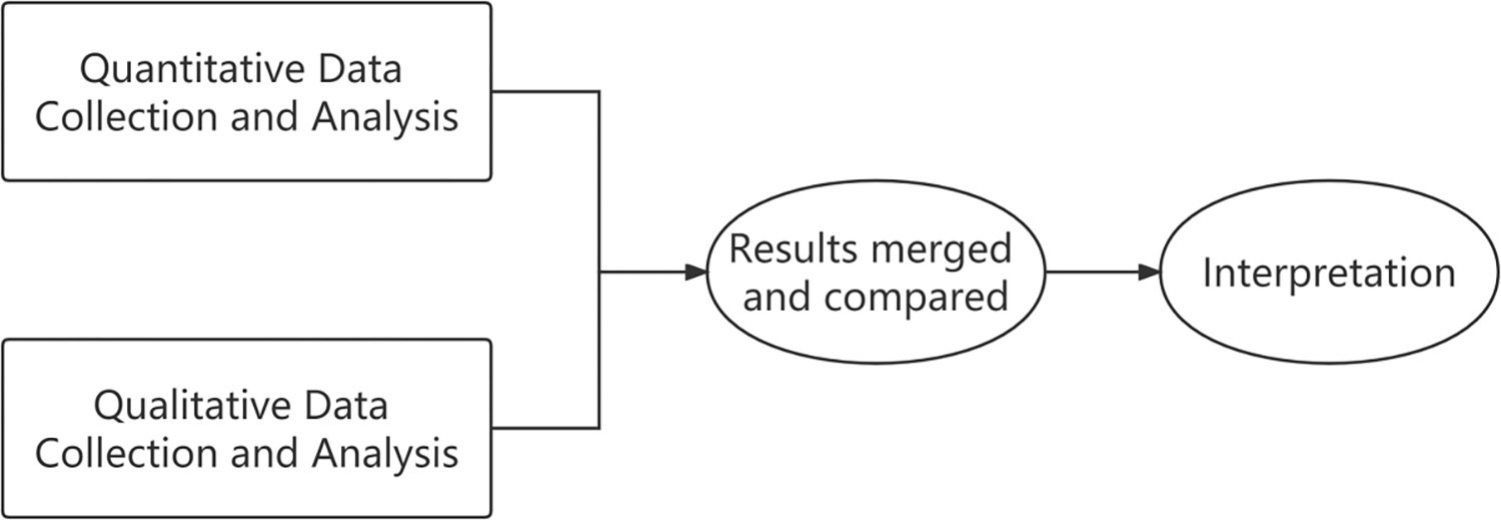
Convergent mixed methods design.

In this study, the researchers employed the convergent design, in which the researcher collected and analysed two separate databases—quantitative and qualitative—and then merged the two databases to compare or comb the results [56]. **Fig 1** is the flow chart of the convergent mixed methods design. Data were collected in two different forms with the intent of obtaining a more complete understanding of the research questions.

Firstly, quantitative data from a large number of teachers were needed to generate an overall picture. However, due to the complex nature of IFLT, it is not enough to gain comprehensive insight by using a questionnaire alone. A semi-structured interview allowed the participants to express their views in their own words, tell stories, or talk about their personal experiences without being limited by answer types. Therefore, the convergent mixed methods design, using both quantitative and qualitative data, provide the potential to explore a fuller and more meaningful and comprehensive understanding of the research questions than if either data has been used alone.

### Research instrument

A questionnaire was used to collect the data from Chinese primary school EFL teachers. The questionnaire is written in Chinese and English to ensure that participants understand every question. The questionnaire design refers to Sercu and Bandura’s instrument and is shaped to be more appropriate for primary school EFL teachers in the Chinese context [23]. It is a questionnaire that contains six parts with 28 items. In this article, the researchers only focus on the parts related to the research questions listed above. There would be a consent form before the online questionnaire began. By ticking the box YES, the participants were providing consent. At the end of the online questionnaire, the researchers asked those willing to participate in the following interviews to leave their contact information. For those willing to participate in the interview, an additional participant information sheet would be provided before the interview and/or classroom observation, and a hard copy of the consent form was obtained before the interview started. Finally, ten teachers were interviewed. The Ethical Committee of the University of Wales Trinity Saint David approved the whole project.

### Participants

The researchers recruited participating teachers through the *Primary Teacher Education Alliance*. Universities and education administrative departments initiated the *Primary Teacher Education Alliance* in central China. One hundred fifty-six primary schools have joined the alliance, ranging from elite urban primary schools to backward rural ones. The researchers used a snowballing sampling method to recruit the participants. Questionnaires were first sent to the liaisons of each school, and they were asked to distribute the questionnaires to the EFL teachers in their schools. Teachers who received the questionnaire can also forward it to primary school English teachers they know. The researchers received a total of 97 questionnaires, of which 93 were valid, which constituted the quantitative data source of this study. The demographic information about the 93 participants is listed in Table 1, and school information is listed in Table 2.

**Table 1:** Demographic information of participants.

Demographics		Frequency	Percentage
**Gender**	Male	13	13.98%
	Female	80	86.02%
**Year of Birth**	1970–1979	4	4.39%
	1980–1989	23	25.27%
	1990–1999	60	65.93%
	2000–2001	4	4.39%
**Degree**	College degree	4	4.30%
	Bachelor degree	81	87.10%
	Master degree	6	6.45%
	Doctoral degree	2	2.15%
**Years of Teaching English**	≦2 years	33	35.48%
	2–5 years	27	29.03%
	6–10 years	23	24.73%
	11–15 years	3	3.22%
	16–20 years	3	4.03%
	> 20 years	4	15.05%
**Teaching Hours per week**	≦ 10 hours	45	48.39%
	11–20 hours	45	48.39%
	21–30 hours	2	2.15%
	31–35 hours	1	1.08%
**Number of Students**	≦ 100 person	43	46.74%
	100–200	34	36.96%
	201–300	11	11.96%
	301–400	3	3.26%
	401–500	1	1.09%

Note

1. Among the 93 people, one said he/she was born in 2006 (15 years old) and another in 2021; these data were considered unreasonable and were excluded.

2. One respondent said the number of students he/she is in charge of is 1000. Even considering China’s huge population base, this number is considered distorted, so it was eliminated from the analysis.

**Table 2:** School information.

School Information		Frequency	Percentage
**School Size**	Under 500	29	31.18%
	500–1000	26	27.96%
	more than 1000	38	40.86%
**School Location**	Urban school	33	35.48%
	Suburban school	23	24.73%
	rural school	37	39.78%
**Socioeconomic level**	Mostly lower income	20	21.51%
	Lower and middle income	38	40.86%
	Mostly middle income	25	26.88%
	Middle and upper income	7	7.53%
	Mostly upper income	3	3.23%

Among the 93 primary schools EFL teachers, 86.02% were female, and 13.98% were male. The majority of them (70.32%) were young teachers under the age of 35. More than half (n = 60, 64.51%) of the teachers were novice teachers with less than five years of teaching experience. Of all the primary EFL teachers, 87.1% had a Bachelor’s Degree, 6.45% had a Master’s Degree, and 2.15% had a Doctoral Degree. Regarding their teaching load, 96.78% taught under 20 hours per week. Most teachers (83.69%) were required to teach around 200 students. Two hundred students might be surprising for counterparts in Western countries, but the average class size in China is 40–70 students due to its huge population. Two hundred students in total means a teacher is in charge of 3–4 classes on average, which is quite reasonable in the Chinese context.

The school information presented a more balanced image. Nearly half of the teachers (40.86%) come from big schools with more than 1000 students. Teachers were balanced distributed in urban/ suburban/ rural schools, providing a good chance for the researcher to get an overall picture. 40.86% of teachers describe their school community as mostly lower and middle income, but a considerable number of teachers come from mostly lower-income (21.51%) and mostly middle-income communities (26.88%).

Ten teachers accepted the researchers’ interview. The researchers identified them as T1 to T10 to protect their privacy. The personal information of the ten interviewees is shown in Table 3.

**Table 3:** Personal information of ten interviewees.

Identifier	Age	Teaching Experience	Educational Background	School Community
**T1**	22	0.5 year	Bachelor’s degree	county
**T2**	46	23 years	Bachelor’s degree	county
**T3**	22	0.5 year	Bachelor’s degree	Provincial capital city
**T4**	44	22 years	Bachelor’s degree	county
**T5**	30	7 years	Bachelor’s degree	city
**T6**	34	12 years	Bachelor’s degree	Provincial capital city
**T7**	22	1 year	Bachelor’s degree	Provincial capital city
**T8**	25	5 years	Bachelor’s degree	county
**T9**	25	4 years	Bachelor’s degree	Rural area
**T10**	24	2 years	Bachelor’s degree	Rural area

Ten interviewees share the same educational backgrounds. They all hold bachelor’s degrees. With ages ranging from 22 to 46 years old, teaching experience varies from half a year to 23 years. In total, four teachers come from cities, six from rural areas, and four based on county.

### Data collection and analysis procedures

The questionnaire was sent to the liaison in each primary school, who would send the link to EFL teachers in their schools. The questionnaire was pilot-tested with a convenient sample of 3 EFL teachers from different primary schools. One of them had been an EFL teacher for three years and is now a doctorate student in English Language Education. Such an educational and working background makes her suggestion to the point. Some changes were made. Then the formal questionnaires were sent out.

Quantitative data were computer-coded, and the SPSSAU (V20.0) online data science algorithm tool was used to analyze data. Both descriptive statistics and inferential statistics were used. The specific statistical methods used will be reported in the findings and discussions.

Interviews were conducted using an interview guide to ensure all relevant topics were covered and allowed free exploring topics that came up through the interview process. Thematic analysis was used to code and analyze qualitative data. The researchers followed the six phases that Braun and Clarke proposed to create meaningful patterns [58]. The six phases are: familiarizing with data, generating initial codes, searching for themes among codes, reviewing themes, defining and naming themes, and producing the final report. The first author is the primary transcriber and coder. The first phase, familiarization with data, occurred through transcription as the researchers listened to the interviews and transcribed the conversation. Once all interviews were transcribed, the information was divided up into domains to build up relevant themes. These domains were created from the interview guide questions based on the research questions.

## Results

### Teachers’ degree of preparation for IFLT

According to the *Curriculum Standards*, English subject is expected to cultivate students’ intercultural competence, and there are specific grading standards for children of different ages to achieve in different compulsory education stages. Teachers were asked to indicate how well prepared they are to meet the *Curriculum Standards* in terms of intercultural teaching, including helping students to form intercultural awareness and to increase their sensitivity to cultural differences, which in turn improves their intercultural communicative competence. Table 4 provides teachers’ responses to these questions.

**Table 4:** Teacher preparedness in meeting the national standards -by frequency and by mean score.

Questions	Choices	Frequency	Percentage	Cumulative percentage	Mean Score
As far as humanity is concerned, the English course undertake the task of improving students’ comprehensive humanities, that is, students can broaden their horizons, enrich their life experiences and form intercultural awareness through English courses. How well prepared are you to meet the standards in this respect?	inadequately prepared	15	16.13	16.13	2.215
	not well prepared	48	51.61	67.74	
	adequately prepared	25	26.88	94.62	
	very well prepared	5	5.38	100.00	
In the initial stage of English learning, students should have a rough understanding of the similarities and differences between Chinese and foreign cultures. In the higher stage of English learning, students should be exposed to foreign cultures to help them expand their horizons and increase their sensitivity to cultural differences, which in turn improve their intercultural communicative competence. How well prepared are you to meet the standards in this respect?	inadequately prepared	11	11.83	11.83	2.290
	not well prepared	49	52.69	64.52	
	adequately prepared	28	30.11	94.62	
	very well prepared	5	5.38	100.00	
Total		93	100.0	100.0	

In general, teachers are poorly prepared for the humanistic aspects of English language education and the enhancement of students’ intercultural competence through English education. We can see from Table 4 that up to 67.74% of teachers choose either inadequately prepared or not well prepared for the first question, and 64.52% of teachers have the same choices for the second question. Whether we judge from the humanity nature of English courses or from the perspective of improving pupils’ intercultural sensitivity, the data showed the same results. This unpreparedness can also be seen in the mean score. The researcher assigned different scores to the four answers, namely inadequately prepared (1), not well prepared (2), adequately prepared (3) and very well prepared (4). The mean scores for the two questions were 2.215 and 2.290, respectively, which shows that teachers are not well prepared for both cultural teaching goals. The median scores for the two questions also reflect teachers’ unpreparedness from another statistical perspective.

Another two questions to indicate teachers’ preparation are the main ways in which they acquire intercultural knowledge and the amount of time they currently spend on expanding their intercultural knowledge and skills per week. Tables 5 and 6 show the results.

**Table 5:** Teachers’ main ways in acquiring intercultural knowledge.

Response Rate and Penetration Rate			
Ways	Response		Penetration Rate (n = 93)
	n	Response Rate	
Reading newspapers, magazines or journal articles about intercultural competence	52	31.90%	55.91%
Watching movies and TV series home and abroad	79	48.47%	84.95%
Communication between colleagues and friends	18	11.04%	19.35%
Seminars or symposiums	7	4.29%	7.53%
Not very interested in and do not devote much time to this area	7	4.29%	7.53%
**Total**	163	100%	175.27%

Chi-Square Goodness-of-Fit Test: χ^2^ = 124.331 p = 0.000

**Table 6:** Time devoted to self-improvement in intercultural knowledge/skills.

Question	Option	Frequency	Percentage (%)	Cumulative Percentage (%)
The amount of time you yourself currently spend each week on expanding your intercultural knowledge/skill are:	Not interested in this area at all	12	12.90	12.90
	Less than one hour	53	56.99	69.89
	1–2 hours	15	16.13	86.02
	More than 2 hours	13	13.98	100.00
Total		93	100.0	100.0

Teachers’ main ways of acquiring intercultural knowledge are ‘watching movies and TV series home and abroad’ (84.95%) and ‘reading newspapers, magazines or journal articles about intercultural competence’ (55.91%). This shows that most teachers do not regard the acquisition of intercultural knowledge as a serious matter. The main way they acquire intercultural knowledge—watching movies and TV series—is more like a means of entertainment rather than a means of formal learning. A similar conclusion can be drawn from the percentage of teachers who participated in relevant seminars or symposiums as a main way to get intercultural knowledge. The number was 7.53%, only 7 out of 93 teachers chose this option.

The picture would be more comprehensive if we take a look at the time teachers currently spend on expanding their intercultural knowledge/skills per week (Table 6). Around 57% of teachers spent less than one-hour acquiring intercultural knowledge and skills. Teachers who spend one hour or more in this area account for around 30%. Such a result implies that although teachers honestly admit that they are not well prepared for implementing IFLT, they do not regard it seriously, since most of them do not devote much time to improve themselves in this regard.

### Affecting factors of teachers’ implementation of IFLT

Teachers reported that they were not well prepared for the implementation of IFLT. Naturally, one may wonder about the reason behind such a situation. In the next question, the researcher asked those teachers what the reasons are for choosing not to spend more time in IFLT, the data obtained are shown in Table 7.

**Table 7:** Reasons for not devote more time to IFLT.

Response Rate and Penetration Rate			
Reasons	Response		Penetration Rate (*n* = 93)
	*n*	Response Rate	
Lack of time in the class	41	13.80%	44.09%
Lack of time for preparation	18	6.06%	19.35%
Shortage of suitable resources	55	18.52%	59.14%
Lack of training in Intercultural Competence myself	54	18.18%	58.06%
Poor knowledge of foreign culture	39	13.13%	41.94%
Class too big	13	4.38%	13.98%
Class too diverse	37	12.46%	39.78%
Lack of management support	11	3.70%	11.83%
Students’ lack of interest	8	2.69%	8.60%
the disconnection between examination requirements and ‘intercultural’ learning objectives	21	7.07%	22.58%
Total	297	100%	319.35%

Chi-Square Goodness-of-Fit Test: χ^2^ = 94.616 *p* = 0.000

As can be seen from Table 7, the four options with the highest response rate are ‘shortage of suitable resources’ (59.14%), ‘lack of training in intercultural competence myself’(58.06%), ‘lack of time in the class’(44.09%) and ‘poor knowledge of foreign culture’(41.94%). Among the four most frequently selected reasons, ‘lack of time in the class’ is the third reason, while this was the most often chosen reason in Sercu and Bandura’s investigation [23]. What is more, this number is significantly lower than the top two reasons. It can be inferred that lack of time in class is not the main reason why teachers cannot carry out intercultural teaching in the Chinese context. The remaining three main options can be roughly divided into two categories: insufficient teaching resources and insufficient training in intercultural competence for teachers.

The researchers also interviewed teachers about the main factors that affect their implementation of IFLT. In this way, teachers could offer answers without being limited by the options provided in the questionnaire. From the qualitative data analysis results, textbooks, the current evaluation system, teachers’ lack of literacy in intercultural competence, insufficient teacher training on intercultural competence, and lack of time are the five main factors that affect teachers’ implementation of IFLT.

The first and most frequently mentioned factor is textbooks. When selecting supplementary cultural materials, assigning homework, or organizing after-class activities, the first factor that teachers consider is whether the material chosen or activities organized are related to the textbooks:

*First of all, it has to be related to our textbooks. It is necessary to consider not only the cultural content but also the language points. For example, the text of The Four Great Inventions is an article written in the past tense. The main language point of this unit is the usage of the past tense, so when we choose reading materials for students, we consider the cultural content as well as the language knowledge. (T3)*

*It should be related to the content of this unit. The words and sentence patterns in it can match the articles in the textbooks, that is the best. (T4)*

It can be seen that textbooks are the first consideration for teachers. It can even be said that textbooks play a leading role in teachers’ teaching. Therefore, if the cultural presentation or intercultural content in the textbook is insufficient, it is easy for teachers to think that intercultural competence is not important. In fact, such a view was also reflected in the interview:

*The textbook does not focus on culture at all. It focuses on the function of the language…to know this word, to know this sentence pattern…to be able to achieve basic communication. The design of the textbook does not pay attention to the culture…I think it does not pay attention to it, at least in the third and fourth grades. It is just a basic communication of words and sentences. (T5)*

An important reason why this teacher did not deliberately carry out intercultural teaching in his/her teaching was that he/she believed that textbooks did not pay attention to the cultural aspect. In contrast, language knowledge was the core content of textbooks. Therefore, the teaching focus should be language knowledge. Such views are quite popular among teachers. In the interviews, the vast majority of teachers mentioned the leading role of textbooks in their teaching. Therefore, correspondingly, if the presentation of cultural content in the textbook does not meet teachers’ expectations, they would easily consider textbooks as one of the factors that are restricting IFLT:

*(The biggest difficulty is) textbooks. Because our teaching are based on textbooks. Everything follows the teaching materials; textbooks are used as a guide. (T5)*

*(The biggest difficulty is) textbooks. Textbooks limit teachers’ teaching. The second is teachers’ quality. Our theoretical knowledge (on intercultural competence) is not enough. (T6)*

The limitation of textbooks on IFLT is also reflected in another aspect, i.e., the current evaluation system. In the current evaluation system, the focus of the evaluation is students’ mastery of the language knowledge in the textbook. Due to the complexity of intercultural competence assessment, the evaluation of students’ intercultural competence is often not the focus or even ignored. Such a tendency also makes teachers pay more attention to the language knowledge in the teaching materials while ignoring intercultural competence intentionally or unintentionally.

*For example, if that article helps me to train my students to read, I might teach, because we have exams after all, right? If you always focus on that (culture), it is not good; we still need to deal with exams. If the article seems to be not very related to the focus of the unit and it is relatively long, I have to spend a lot of time on that article. Then I might just mention it and go on. That’s the way it is. (T2)*

*The biggest difficulty is that our students may still pay more attention to grades. (T3)*

Teachers are talking about the importance of test scores, while the focus of the test is on language knowledge in textbooks rather than (inter)cultural knowledge, which makes both textbooks and the current evaluation system realistic factors that restrict the cultivation of students’ intercultural competence. This environment makes teachers and students unconsciously take language knowledge acquisition as their primary goal in teaching/learning a foreign language.

In addition, teachers’ lack of literacy in intercultural competence is also an important reason why IFLT cannot be successfully implemented. In fact, it is a common phenomenon among primary school EFL teachers to think that they do not have enough knowledge or understanding of intercultural competence:

*I don’t think I am very clear about these things (culture). I’m still at a very shallow level. I just stop at introducing some cultural knowledge to students and let them know there is such a thing. That’s how it is. (T4)*

*Because first of all…Although I am an English teacher, we are also in a kind of… . when it comes to foreign countries…actually I don’t know much… . In fact, for all of us, we have a relatively shallow understanding of their culture, so when we teach students, very deep cultural exchange does not exist, I think. It is just about language structure…very simple…just language level, it doesn’t involve culture. (T5)*

Those quotes were consistent with teachers’ unpreparedness for IFLT in the quantitative data. Teachers believe that they have insufficient literacy in intercultural competence, so they can only carry out cultural teaching in a relatively simple way. To promote the implementation of IFLT, teachers’ IFLT ability is very important. This ability includes not only teachers’ understanding of other countries’ cultures, but also teachers’ ability to design and implement intercultural teaching based on the characteristics of cultural teaching as well as teaching materials. The improvement of teachers’ IFLT ability is far from enough to rely only on teachers’ self-study. Teachers also realized this problem too. Many teachers mentioned insufficient education and training in intercultural competence:

*I don’t know what kind of activities can really promote students’ intercultural competence. In the past, including in university, my teachers may have mentioned this aspect, but I still lack deep understanding. (T1)*

*I still don’t know how to cultivate children’s intercultural competence systematically and consciously. In fact, I think that as teachers, we receive relatively little education and training in this area. (T7)*

As can be seen from the above introduction, whether in school or in in-service training, teachers thought they had not received enough education and training in intercultural competence, so they did not know how to systematically cultivate students’ intercultural competence. This leads to random and fragmented cultural teaching observed in many studies [11, 12]. Hence, we can say that training on teachers’ IFLT ability should be a focus of teacher education. The current frequency of training on teachers’ intercultural competence cannot keep up with the requirement of the times.

Finally, teachers generally lack time and energy for self-improvement in any form. At present, heavy tasks and many trivial affairs for front-line teachers is a quite common phenomena in China. The impact of teachers’ lack of time and energy is mainly reflected in two aspects. First, teachers do not have enough time to dig deep into the cultural content of textbooks, so language knowledge as the focus of teaching has become a ‘shortcut’; Second, teachers do not have time for self-improvement. As a result, it leads to teachers’ lack of literacy in intercultural competence:

*Because I taught in rural school before, at that time it (English) was not a major subject. I also taught mathematics at that time, I did not have the time and energy to dig deep into the textbooks of English, so I would only focus on language itself. (T5)*

*There are actually quite a lot of online training courses, but indeed…we have very heavy tasks, some required online training courses…to be honest… we do not have time to (watch). We have to spend a lot of time preparing lessons, making PPT, grading the papers for students… . many things. Training is supposed to be a good way…to improve…I think it is really good if you have time to listen, but the reality is that we don’t have time. In addition, we don’t have the energy to learn. I can’t devote myself to learning. It takes time and energy to study. (T4)*

From the above quotes, we can see that lacking time and energy has restricted the improvement of teaching quality, and has also become an important reason for restricting the implementation of IFLT. In the interview, we can feel that most teachers are aware of their lack of preparation for implementing IFLT, but due to the constraints of practical conditions, even if they recognize the problem, they do not have the time or energy to improve themselves. This also helps explain why up to 73.12% of teachers spend less than two hours per week on improving intercultural knowledge/skills even though they clearly understand their unpreparedness. How can we enable teachers have enough time to prepare for the class is an important issue that need to be addressed in China.

To conclude, from teachers’ perspective, their literacy in intercultural competence is generally not enough, and they lack time and energy for self-improvement. They are far from adequately educated and trained in intercultural competence. In addition, the textbook and the current evaluation system are also two factors restricting IFLT.

### Support needed to implement IFLT

We have now identified factors restricting the implementation of IFLT, which answered RQ2. The next question we want to resolve is how we can help teachers implement IFLT. In other words, what support do teachers need to achieve the transformation in foreign language education? As noted by many studies, it is necessary to develop intercultural competence in pre-service and in-service teachers education systematically [22, 59, 60]. Therefore, the researchers investigated what experiences were the most helpful in preparing teachers for future intercultural foreign language teaching. ‘Independent study/reading’ and ‘travel and living in other culture’ were the most highly rated among the options provided (see Table 8), with a mean score of 2.398 and 2.376, respectively. The option ranked third is ‘more support/open attitude from school or district administrators’ (2.344). The high rating of this option indicates the importance of supports from education administrators. The transformation from traditional language teaching methods to IFLT requires more than intercultural knowledge, such a transformation is a systematic project includes the change of language learning objectives, the classroom activities, the language teaching methods and assessment etc. While one cannot expect any individual teachers can accomplish such a transformation with his/her own efforts. Teachers obviously realized that too, therefore, more support from school got a very high rating.

**Table 8:** The helpfulness of different experiences.

Different experiences	Mean score
Independent study/reading	2.398
Travel and living in other countries	2.376
More support/open attitude from school or district administrators	2.344
Planning time to work on your own	2.333
Training courses work in the knowledge of intercultural communication	2.301
Planning time to work with teachers of other disciplines	2.290
Training courses work in the nature of culture	2.258

Another surprising finding is that training courses on culture or intercultural communication got very low rates from teachers (2.301 and 2.258 respectively), ranked the first from last and the third from last. Before the data analysis, we thought that the training course on intercultural competence would be very helpful for teachers who would like to promote IFLT, but the data showed that teachers thought exactly the other way around. We could gain some insights into such a result from another question in the questionnaire. Teachers were asked whether there are any programs or contents related to the cultivation of intercultural competence in the in-service teacher training they have attended. Up to 66.67% (see Table 9) of teachers gave positive answers to this question, which means that in the teacher training programs they have participated in, there was content related to intercultural competence, whether it be in every/most or some training program. If we combine the two data, we can infer that teachers think training courses are not helpful, because in the training courses they have attended, the content related to intercultural competence does not help their teaching in the real context. If such an inference holds true, then there must be some problems with the current intercultural competence training courses. The training curriculum may be unreasonable, or the training content cannot guide actual teaching. No matter what the problems are, those problems lead teachers to conclude that intercultural competence training is of little use. We shall discuss this point with the qualitative data again.

**Table 9:** Teacher training programs related to intercultural competence.

Question	Options	Frequency	Percentage	Cumulative Percentage
In the in-service teacher training program you have attended, are there any program or contents related to the cultivation of intercultural competence?	Yes, in every training program	11	11.83	11.83
	Yes, in many training program	22	23.66	35.48
	Yes, in some training program	29	31.18	66.67
	No, only in a few training program	19	20.43	87.10
	No, not at all	12	12.90	100.00
Total		93	100.0	100.0

We also asked teachers about the helpfulness of materials in promoting their future IFLT. Table 10 shows the results. English materials on the target culture and intercultural communication were the ones teachers thought would be the most helpful, while general materials on culture was rated as the least helpful. The respondents are EFL teachers in primary schools, so it can be understood that they think English materials on English cultures would be most helpful in their future teaching. It would be interesting to investigate why most teachers think general materials on culture are of little help. But from such a result, we can infer that teachers still put the target culture (English in this study) as the focus of their cultural teaching while ignoring the imparting of culture-general knowledge.

**Table 10:** The helpfulness of different cultural teaching materials.

Different materials	Mean score
Materials on the target culture and intercultural communication, written in English	2.301
Materials on the target culture and intercultural communication, written in Chinese	2.290
General materials on culture (i.e., not on specific cultures)	2.247

Teachers’ answers in the interviews corroborated with the quantitative data. The support teachers need can be divided into three categories. The first category is support from the external environment, for example, support from authorities and school administrations; the second type is materials and resources related to intercultural teaching; the third type is teacher training on intercultural competence. Interestingly, teacher training is the most frequently mentioned support of the three types of support needed, contradicting the quantitative data. In the quantitative data results, training courses working on the nature of culture and intercultural competence got a low ranking (see Table 9). However, teacher training is a need mentioned by almost all teachers in the interviews. The contradiction of the results might lie in the training content. From the interview data, we can tell that teachers prefer practical and methodological training. A few teachers mentioned training in theoretical frameworks, but more teachers need training in methods, that is, training in ‘how to’:

*I’m now very urgent to know the background and definition of this concept. Secondly, I want to have something that can be practiced… . some experience… . or methodology. How to accomplish such a task? A practical method. Use a lecture to clarify its definition and then tell us how it can be cultivated, what methods are effective, or what research or practice has been done. Demonstration classes are also very good; they are more direct. And in such training, it is best to give us some practice opportunities. Guide me after the practice. This training would be more meaningful to our daily teaching. (T9)*

*I need an experienced person to guide me on how to carry out such activities. I have never conducted this kind of activity; I have no experience in this regards. It (Training) should be able to provide me with a systematic process of organizing teaching. For example, if I want to do that, what preparations and various equipment do I need, and what are the basic procedures? I don’t understand this concept, and I don’t understand how to carry out activities that can promote students’ intercultural competence…I have no idea. (T10)*

*First of all, I definitely want to supplement my intercultural competence knowledge. After I understand what it is, I would like to receive training on how to cultivate children’s intercultural competence in my daily teaching. So (training on) teaching methods may be more useful. The demonstration lessons or the discussion of teaching cases. (T8)*

As seen from the above quotes, teachers are very willing to teach intercultural competence and keen to spend time learning and practicing. Most front-line primary school EFL teachers do not receive scientific research training; therefore, it is unrealistic to rely on their teaching experience and intuition to try out which teaching methods can promote students’ intercultural competence. An efficient solution should be to regularly provide teachers with IFLT ability training and pass on the teaching methods that have been proved to be effective by academia. The training content should focus on the interpretation of intercultural teaching principles and the guidance of teaching methods, including the design and presentation of cultural content in the teaching materials.

The second type of support is external environment support, which mostly appeared in the interviews with rural EFL teachers. Rural primary schools usually have a backward and poor learning environment for English, and their school infrastructure and support from parents are not as good as urban primary schools. Rural students’ English proficiency is generally weaker than that of urban pupils. Therefore, for EFL teachers in rural areas, the environment is not conducive to implementing IFLT. This is the most realistic factor as well as the one that needs to be improved urgently in rural areas:

*The biggest difficulty is the lack of support. In our school, the only opportunity for students to speak English is in English classes. They do not speak English at other places or at other times. Many of them cannot listen or speak in English, not to mention intercultural competence… . I don’t know how to start at all. I have some friends who are EFL teachers in cities. The support they receive, for example, if they want to hold an event, the venue, the equipment, and the support from parents. These are all different, totally different. (T10)*

The third type of support needed is the construction of related resources. Relevant resources include two aspects. The first is building of teacher groups or Communities of Practice, as Wenger put it [61]. Many teachers have mentioned that relying on individuals to implement IFLT is unrealistic. It requires teamwork, with each teacher doing their own part. However, such division and cooperation are difficult to achieve in many small-sized primary schools. The second aspect is the materials and resources related to intercultural teaching, including textbooks and other available resources. The textbook is the major concern for most teachers when designing their classroom teaching. Therefore, constructing intercultural resources related to textbooks and other cultural teaching materials is indispensable if we want to promote the development of IFLT. Suppose a shared resources library can be established. In that case, it can firstly improve teachers’ awareness and willingness to IFLT and make teachers realize that intercultural competence is one of the important goals of foreign language teaching. On the other hand, it can reduce teachers’ workload on searching and choosing resources; hence, more time could be devoted to teaching design and implementation, improving teaching quality.

*Lack of partner. For example, my school has only two English teachers, and we have to teach all the students in four grades. If you want to do something about intercultural competence, you must design it carefully, using project-based learning or similar methods. If you only use the current textbooks and follow the content in the textbooks, you definitely will not be able to achieve this. So you have to search for materials and resources, sort out the texts you need……Only two of us can not do all those work…and to be honest, our literacy in this regard… .is just so-so. So there is a lack of professional support and the support from working partners. (T10)*

*I hope this course teaches me how to cultivate children’s intercultural competence. And it can provide me with some…for example, ideas, good materials and resources. Tell me what kind of ability needs to be developed and use what materials to cultivate. (T7)*

In a word, promoting IFLT is a long-term and complex task, which requires improving teachers’ intercultural literacy and support from educational departments, school administrations, and the construction of related resources. One teacher nicely sums up the support they need in his/her interview:

*Intercultural competence can be cultivated (in primary schools), but only in an ideal environment. It requires many conditions… .it needs a good environment, good teaching materials, and teachers’ good professional ability. (T9).*

## Discussion

In conclusion, most Chinese primary school EFL teachers describe themselves as not well prepared for IFLT. But paradoxically, they do not spend much time on self-improving in the IFLT area. Teachers’ main way to improve their intercultural knowledge is more like a means of entertainment rather than formal learning. Only 4.29% of teachers regard it as a serious matter—they would attend relevant seminars or symposiums. Combining quantitative and qualitative data, we conclude that teachers’ lack of intercultural competence literacy and the lack of suitable sources are two major factors that constrain the implementation of IFLT. More support/open attitudes from school or district administrators echoed with teachers’ responses in the interview that more external support is expected. Furthermore, teacher training and the construction of intercultural teaching materials echoed teachers’ illiteracy in intercultural competence and the lack of suitable resources in IFLT. Overall, the quantitative and qualitative data in this study were mutually supportive, ensuring the reliability of the findings.

Several interesting findings need further discussion. Firstly, in a similar study conducted in Europe, teachers chose the lack of time in class as the biggest reason for not implementing intercultural teaching [23]. However, many Chinese EFL teachers regard textbooks as the biggest difficulty in intercultural teaching, which is unique in the Chinese context. In China, textbooks are regarded as the core of the curriculum. This situation, coupled with the tradition of emphasizing the teacher and the textbook, has accentuated the influence of the textbook on foreign language learning patterns [15], making foreign language teaching a ‘practice defined by the textbook’ [62]. This is evident in the interviews with teachers. The fact that some teachers regard textbooks as the biggest difficulty in implementing IFLT conformed with previous studies where they found that the coursebooks available are inappropriate for achieving IFLT objectives [51, 52, 55].

The main purpose of this study was not to investigate the position of textbooks in IFLT, so the researchers did not go into depth how teachers understand and evaluate the cultural representation in textbooks. But other research in this area has shown that studies on the role of textbooks in IFLT are indeed far from enough [15]. Zhu [63] reviewed the literature on IFLT in 15 Chinese foreign language journals and found only two research papers evaluating intercultural foreign language teaching materials over a ten year period, indicating that research on IFLT materials in China has lagged slightly. Furthermore, many studies have also shown that the choice of cultural topics in the textbook needs to be improved in both breadth and depth. The presentation of cultural content is fragmented and remains at the superficial level of the tourist’s perspective [64, 65]. In addition, a great proportion of cultural content in the textbook is cultural knowledge related to the inner circles of English-speaking countries [15, 49, 51], which echoes the call by scholars for more inclusion of other countries’ cultures in English teaching materials [53].

The lack of research into the intercultural content of textbooks is one aspect, and on the other hand, research into the interaction between teachers and teaching materials is even less. American and Tajabadi’s research revealed teachers’ dissatisfaction with the cultural content and presentation of culture in the textbooks [52]. While our findings suggest that teachers’ evaluation of textbooks influence teachers’ willingness and practices of IFLT. Therefore, one of the directions for future research is what intercultural content teachers expect, how this content is presented in the teaching materials, and how teachers deal with that content in class.

Second, teachers’ ranking of the helpfulness of different experiences (independent learning/ reading ranked first, followed by travelling and living in other countries, see Table 8) in improving IFLT indicates that they might have a one-sided understanding of IFLT, which is consistent with previous studies [28–30]. Traveling and living in other countries ranked second in terms of helpfulness in enhancing their future foreign language teaching. And many teachers also expressed similar thoughts in the interviews. They believe that a major reason for their insufficient ability in IFLT is that they have never been abroad and have limited contact with foreign cultures. Hence they do not know much about foreign cultures. These teachers equate having overseas experience with having intercultural competence, However, many empirical studies have shown that intercultural competence does not improve automatically through mere intercultural contact, shallow or negative intercultural experiences may even strengthen stereotypes [35–37]. Intercultural scholars have pointed out that positive attitude change through intercultural experience requires reflection on unusual events [66, 67]. Moreover, there were also studies proving that teachers’ intercultural awareness could be improved without direct, overseas experience [68, 69], which opposed teachers’ opinion that intercultural competence can only happen with a direct connection with other cultures. Other scholars also agree that mere exposure to another culture does not necessarily mean a higher degree of intercultural competence. The media, education, and other forms of indirect contact can also provide opportunities to gain knowledge and understanding of other nations and cultures [70]. This is particularly valuable for teachers from countries or areas where economic conditions do not allow traveling or training abroad.

Third, general materials on culture were considered the least helpful among all teaching materials (see Table 10), which shows that in actual teaching, teachers pay more attention to the target language’s culture while ignoring the general cultural knowledge. In other words, teachers’ understanding of IFLT is still at the stage of ‘the foreign-cultural approach’ [71], under which cultural knowledge is mainly the ‘big C’ of the target culture, such as historic figures and major historical events [65]. Such a finding conformed with previous studies conducted in different contexts, where they found that teachers lack an understanding of intercultural competence, and that most of their cultural teaching remains country-centered under the influence of an essentialist view of culture [29–31]. Compared with ‘the foreign-cultural approach’, IFLT requires teachers put more emphasis on general cultural knowledge. Sun and Bennett likened general cultural knowledge to a ‘cultural map’, like a software system for intercultural communication [72]. Students can teach themselves specific cultural knowledge when installing such a software system. Sun also pointed out that focusing only on specific cultural knowledge in foreign language teaching is a practice of ‘seeing the trees but not the forest’. A student with only specific cultural knowledge cannot conduct independent intercultural exploration when he/she gets lost in the cultural forest. IFLT should go beyond explaining and reciting specific cultural knowledge; it should guide students to learn the general theories and methods of cultural analysis and use them to solve specific problems in intercultural communication. In this sense, general materials on culture should be more helpful than materials on target culture. But teachers’ responses in this study indicate that they do not fully understand the connotation of IFLT. However, we must state clearly that this is not to say that specific cultural knowledge is not important, but in IFLT, we aim to develop general intercultural competence that allows students to cross different cultural boundaries freely. The ideal way of IFLT is combining the study of specific cultural knowledge with the study of general cultural knowledge and understanding the general principles of intercultural communication through specific cases.

In the end, it is worth backing to teacher training again here. From the above discussion, we concluded that most Chinese primary school EFL teachers have a one-sided understanding of IFLT. Therefore, teacher training in intercultural competence is not only a need raised by teachers themselves, but also an indispensable part to further develop IFLT. Training teachers in intercultural competence has been a long-standing call in the academic community. Scholars have examined the effectiveness of various training programs from different perspectives and proposed different designs of training methods [44, 45]. Compared to previous top-down validation studies by scholars, our study offers a new line of research: to listen to the needs of front-line teachers and design training programs from the bottom up. In general, front-line teachers preferred ‘hands-on’ training, with more emphasis on how to. Most of the teachers interviewed said they would like training in teaching methods and tools. As for the training form, many of them mentioned demonstration classes, and few mentioned theoretical training. These were answers gained from teachers’ perspectives. However, theoretical training on relevant concepts such as culture, language, intercultural competence and IFLT is still necessary from the researchers’ perspective. As discussed above, teachers’ understanding of these concepts cannot meet the requirements raised by IFLT, and they did not realize the importance of theory training. Hence, training in the theoretical parts can be moderately reduced, but it should not be absent.

The gap between current training courses in intercultural competence and what teachers expect from them explained the contradictory results of the quantitative and qualitative data regarding the helpfulness of intercultural training courses in this study. On the one hand, teachers doubt the helpfulness of current intercultural training courses, but on the other hand, they urgently want to improve their IFLT competencies through training courses. Such a contradiction suggests that we need more bottom-up research to optimize the current training courses and listen more to the needs of front-line teachers, which is both one of the future research directions and the research significance of the current study.

## Conclusion

To summarize, primary school EFL teachers generally felt unprepared for the transformation to IFLT. More specifically, their approach to acquiring intercultural knowledge is informal, and they spend limited efforts on improving themselves in intercultural knowledge. Second, teachers’ lack of literacy in intercultural competence and the lack of suitable resources to teach about intercultural competence, coupled with the current evaluation system, teachers’ lack of time constrain the implementation of IFLT. Accordingly, more support from the external environment, construction of related resources, and teacher training courses on intercultural competence are solutions to help teachers become better prepared for IFLT.

Based on these findings, the discussion part focused on the role of textbooks, experiences abroad, and general materials on culture in developing IFLT. Most foreign language teachers internationally consider lack of classroom time to be a major impediment to IFLT, whereas most Chinese primary school EFL teachers see the textbook as an impediment to IFLT, which is relatively rare in other similar studies and is one of the main findings of the current study. The discussion of the role of experience abroad and the general materials on culture in IFLT reflects the gap between the perceptions of academics and front-line teachers. Such a gap, coupled with teachers’ low ratings of current intercultural training courses and teachers’ preference for practice-oriented training courses, suggests that we should listen more to the voices of front-line teachers. This is how research findings can be truly translated into pedagogical change.

Accordingly, the implications of this study are two-fold. First, it indicates that different countries may face different difficulties in implementing IFLT, which requires domestic researchers to tailor their studies to the national context. In the current study, the hindering effect of textbooks on IFLT is rarely seen in previous studies. Although researchers have long studied the important role of foreign language teaching materials, there is still a lack of research on how teachers perceive the intercultural content in textbooks and the interaction between teachers and teaching materials. More future research is expected to explore the interaction between teachers and teaching materials and how teachers present the intercultural content in the materials in their classroom. Second, this study shows the importance of ‘hands-on’ training in IFLT. Previous studies on teachers’ intercultural competence training have mostly adopted a top-down research design, whereby the researcher designs a training program by carefully reading the literature, and then validates the effectiveness of the training program through empirical research. This study, however, explores what front-line language teachers expect a training course to look like through a bottom-up research design. The findings of this study can therefore inform future training courses.

The current study investigated Chinese primary school EFL teachers’ preparation for IFLT, its influencing factors and the support teachers need to promote the sustainable development of IFLT by collecting both quantitative and qualitative data. The transformation from traditional skill-oriented language teaching to the more challenging intercultural foreign language teaching requires efforts from researchers, foreign language teachers and educational departments. This study illustrates the need for more voices from front-line teachers in the design of textbook and training program in the Chinese context, and it is hoped that this study will provide inspiration to other countries like China where English is taught as a foreign language.

## References

[1] Dai XD, Guo MC. Intercultural Communication Competence: Conceptualization and Its Development in Cultural Contexts and Interactions. Newcastle Upon Tyne, Uk: Cambridge Scholars Publishing; 2014.

[2] Hammer MR, Bennett MJ, Wiseman R. Measuring Intercultural sensitivity: the Intercultural Development Inventory. International Journal of Intercultural Relations. 2003;27: 421–443. doi: 10.1016/s0147-1767(03)00032-4

[3] Spitzberg BH, Changnon G. Conceptualizing Intercultural Competence. In: Deardorff DK, editor. The SAGE Handbook of Intercultural Competence. London: Sage Publications; 2009. pp. 2–52.

[4] Deardorff DK. Identification and Assessment of Intercultural Competence as a Student Outcome of Internationalization. Journal of Studies in International Education. 2006;10: 241–266. doi: 10.1177/1028315306287002

[5] Byram M. Teaching and assessing intercultural communicative competence. Clevedon: Multilingual Matters; 1997.

[6] Zhang HL. Intercultural Education as a Means of Teaching Foreign languages: History, Present and Future. Foreign Language World. 2012;149: 2–7. (In Chinese)

[7] Li HE. On the cultural character of English curriculum [PhD Thesis]. [Southwest University]; 2012. (In Chinese)

[8] Guo FM. A cultural review on English language education in primary and secondary school. Journal of the Chinese Society of Education. 2011;(09):67–9. (In Chinese)

[9] Maria P. Developing Intercultural Competence in English Language Teachers: Towards Building Intercultural Language Education in Colombia. These. 2018. Available: http://ethese.dur.ac.uk/12619/

[10] Liddicoat AJ. Pedagogical Practice for Integrating the Intercultural in Language Teaching and Learning. Japanese Studies. 2008;28: 277–290. 10.1080/10371390802446844

[11] Marczak M. New Trends in Teaching Language and Culture. In: Komorowska H, Alesksandrowicz-Pedich L, editors. Coping with diversity: Language and Culture Education. Poland: Wydaxnictwo SWPS Academica; 2010.

[12] Piątkowska K. From Cultural Knowledge to Intercultural Communicative Competence: Changing Perspectives on the Role of Culture in Foreign Language Teaching. Intercultural Education. 2015;26: 397–408. 10.1080/14675986.2015.1092674

[13] Heggernes SL. A Critical Review of the Role of Texts in Fostering Intercultural Communicative Competence in the English Language Classroom. Educational Research Review. 2021;33: 100390. 10.1016/j.edurev.2021.100390

[14] Byram M. Teaching and Assessing Intercultural Communicative Competence: Revisited. Bristol: Multilingual Matters; 2020.

[15] Sun YZ, Liao HJ, Zheng X, Qin SQ. Research on Intercultural Foreign Language Teaching and Learning. Beijing: Foreign Language Teaching and Research Press; 2021. (In Chinese)

[16] Barrett M. Children’s Knowledge, Beliefs and Feelings about Nations and National Groups. Psychology Press; 2013.

[17] Barrett M, Buchanan-Barrow E. Children’s Understanding of Society. In: Smith PK, Hart CH, editors. The Wiley-Blackwell Handbook of Childhood Social Development (2nd edition). Chichester: Wiley-Blackwell; 2011. pp. 584–602.

[18] Powlishta KK, Serbin LA, Doyle A-B, White DR. Gender, ethnic, and Body Type biases: the Generality of Prejudice in childhood. Developmental Psychology. 1994;30: 526–536. 10.1037/0012-1649.30.4.526

[19] Dziedziewicz D, Gajda A, Karwowski M. Developing children’s Intercultural Competence and Creativity. Thinking Skills and Creativity. 2014;13: 32–42. 10.1016/j.tsc.2014.02.006

[20] Michael O, Rajuan M. Perceptions of ‘the Other’ in Children’s drawings: an Intercultural Project among Bedouin and Jewish Children. Journal of Peace Education. 2009;6: 69–86. 10.1080/17400200802658407

[21] Zhang X, Zhou M. Interventions to Promote Learners’ Intercultural competence: a meta-analysis. International Journal of Intercultural Relations. 2019;71: 31–47. 10.1016/j.ijintrel.2019.04.006

[22] Li YH. Intercultural Awareness in Foreign Language Teaching: a Chinese Perspective. Journal of Language Teaching and Research. 2016;7: 768. 10.17507/jltr.0704.18

[23] Sercu Lies, Bandura E. Foreign language teachers and intercultural competence: an international investigation. Clevedon: Multilingual Matters; 2005.

[24] Oranje J, Smith LF. Language Teacher Cognitions and Intercultural Language teaching: the New Zealand Perspective. Language Teaching Research. 2017 Feb 13;22(3):310–29.

[25] Shao S, Chen J. A Survey on the Intercultural Sensitivity of High School English Teachers. Foreign Language Research. 2011;(3):144–7. (In Chinese)

[26] Zhang C. Quantitative Research on the Beliefs of Foreign Language Teachers in Chinese Universities -Based on the Cultivation of Intercultural Communicative Competence. Foreign Languages in China. 2014;(6):91–5, 105. (In Chinese)

[27] Han XH. The Current Situation and Reflections on the Cultivation of Intercultural Communication Skills of Students in Higher Education—Using English Teachers in Higher Education as an Examination Dimension. Foreign Language Research. 2014;(03):106–10. (In Chinese)

[28] Qian LH. Concpetions of the Role of Culture in Foreign Language Education in China. PhD Thesis. 2011.

[29] Maria P-DB. Developing Intercultural Competence in English Langauge teachers: Towards Building Intercultural Language Education in Colombia. PhD Thesis. 2019.

[30] Pattaraworathum N. Culture Teaching Practices of Lower Secondary School EFL Teachers from the Global English Perspectives: A Qualitative Case Study in Thailand. PhD Thesis. 2021.

[31] Munandar MI. Interculturality and Islam in Indonesia’s high-school EFL Classrooms. International Review of Applied Linguistics in Language Teaching. 2022;0. 10.1515/iral-2021-0200

[32] Peiser G, Jones M. The Influence of Teachers’ interests, Personalities and Life Experiences in Intercultural Languages Teaching. Teachers and Teaching. 2013;20: 375–390. 10.1080/13540602.2013.848525

[33] Czura A. Major Field of Study and Student Teachers’ Views on Intercultural Communicative Competence. Language and Intercultural Communication. 2016;16: 83–98. 10.1080/14708477.2015.1113753

[34] Wolff F, Borzikowsky C. Intercultural Competence by International Experiences? an Investigation of the Impact of Educational Stays Abroad on Intercultural Competence and Its Facets. Journal of Cross-Cultural Psychology. 2018;49: 488–514. 10.1177/0022022118754721

[35] Meleady R, Seger C, Vermue M. Evidence of a Dynamic Association between Intergroup Contact and Intercultural Competence. Group Processes & Intergroup Relations. 2020;24: 1427–1447. 10.1177/1368430220940400

[36] Gao YH. Speaking to the World: Who, When and How? An Ethnographic Study of Slogan Change and Identity Construction of Beijing Olympic Games Volunteers. Asian Journal of English Language Teaching. 2010;20:1–26.

[37] Xu L, Gao YH. Attitudes of College Student Volunteers to World English before and after Four Large-Scale International Events. Foreign Language Education. 2014; 39–47. (In Chinese)

[38] Garrido C, Álvarez I. Language Teacher Education for Intercultural Understanding. European Journal of Teacher Education. 2006;29: 163–179. 10.1080/02619760600617342

[39] Han H. An Investigation of teachers’ Perceptions of Culture Teaching in Secondary School in Xinjiang, China. PhD Thesis. 2010.

[40] Maijala M. Culture Teaching Methods in Foreign Language education: pre-service Teachers’ Reported Beliefs and Practices. Innovation in Language Learning and Teaching. 2018;14: 133–149. 10.1080/17501229.2018.1509981

[41] Han XH, Song L. Teacher Cognition of Intercultural Communicative Competence in the Chinese ELT Context. Intercultural Communication Studies. 2011;20: 175–192.

[42] Larzén‐Östermark E. The Intercultural Dimension in EFL‐Teaching: A Study of Conceptions among Finland‐Swedish Comprehensive School Teachers. Scandinavian Journal of Educational Research. 2008;52: 527–547. 10.1080/00313830802346405

[43] Young TJ, Sachdev I. Intercultural Communicative competence: Exploring English Language Teachers’ Beliefs and Practices. Language Awareness. 2011;20: 81–98. 10.1080/09658416.2010.540328

[44] Şalli Çopur D. Integrating Intercultural Communicative Competence into Teacher Education for Young Learners. Cankaya University Journal of Humanities and Social Sciences. 2021;15: 330–347. 10.47777/cankujhss.1047509

[45] Safa MA, Tofighi S. Intercultural Communicative Competence Beliefs and Practices of Iranian pre-service and in-service EFL Teachers. Innovation in Language Learning and Teaching. 2021;16: 1–12. 10.1080/17501229.2021.1889562

[46] Figueredo-Canosa V, Ortiz Jiménez L, Sánchez Romero C, López Berlanga MC. Teacher Training in Intercultural Education: Teacher Perceptions. Education Sciences. 2020;10: 81. 10.3390/educsci10030081

[47] Romijn BR L. Slot P, Leseman PPM. Increasing Teachers’ Intercultural Competences in Teacher Preparation Programs and through Professional development: a Review. Teaching and Teacher Education. 2021;98: 103236. 10.1016/j.tate.2020.103236

[48] Haerazi H, Nunez JL. Promoting Intercultural Competences and Communication Skills through English Textbooks within Multilingual Education. Journal of Language and Literature Studies. 2022;1: 75–82. 10.36312/jolls.v1i2.610

[49] Song B. Exploring the Cultural Content in Chinese ELT Textbooks from Intercultural Perspectives. The Journal of Asia TEFL. 2019;16: 267–278. 10.18823/asiatefl.2019.16.1.17.267

[50] Abdul Rahim H, Jalalian Daghigh A. Locally Developed versus Global textbooks: an Evaluation of Cultural Content in Textbooks Used in English Language Teaching in Malaysia. Asian Englishes. 2019;22: 1–15. 10.1080/13488678.2019.1669301

[51] Minh NTT, Phuong CTH. An Evaluation of the Intercultural Orientation of Secondary English Textbooks in Vietnam. In: Le VC, Nguyen HTM, Nguyen TTM, Barnard R, editors. Building Teacher Capacity in English Language Teaching in Vietnam. London: Routledge; 2019. Available: 10.4324/9780429457371

[52] Amerian M, Tajabadi A. The Role of Culture in Foreign Language Teaching textbooks: an Evaluation of New Headway Series from an Intercultural Perspective. Intercultural Education. 2020;31: 1–22. 10.1080/14675986.2020.1747291

[53] Setyono B, Widodo HP. The Representation of Multicultural Values in the Indonesian Ministry of Education and Culture-Endorsed EFL textbook: a Critical Discourse Analysis. Intercultural Education. 2019;30: 383–397. 10.1080/14675986.2019.1548102

[54] Gedik Bal N. Students’ and Instructors’ Evaluation of a Foreign Language Textbook from an Intercultural Perspective. Journal of Language and Linguistic Studies. 2020;16: 2023–2038. 10.17263/jlls.851032

[55] Smakova K, Paulsrud B. Intercultural Communicative Competence in English Language Teaching in Kazakhstan. Issues in Educational Research. 2020;30: 691–708.

[56] Creswell JW, Plano Clark VL. Designing and Conducting Mixed Methods Research. 3rd ed. Los Angeles: Sage; 2018.

[57] Johnson RB, Onwuegbuzie AJ, Turner LA. Toward a Definition of Mixed Methods Research. Journal of Mixed Methods Research. 2007 Apr;1(2):112–33.

[58] Braun V, Clarke V. Using Thematic Analysis in Psychology. Qualitative Research in Psychology. 2006;3(2):77–101.

[59] Byram M, Risager K. Language teachers, politics, and Cultures. Clevedon, UK; Philadelphia: Multilingual Matters; 1999.

[60] Polat S, Barka TO. Preservice Teachers’ Intercultural Competence: a Comparative Study of Teachers in Switzerland and Turkey. Eurasian Journal of Educational Research. 2014;14: 19–38. 10.14689/ejer.2014.54.2

[61] Wenger E. Communities of Practice. Cambridge University Press; 1999.

[62] Akbari R. Postmethod Discourse and Practice. TESOL Quarterly. 2008;42: 641–652. 10.1002/j.1545-7249.2008.tb00152.x.

[63] Zhu LX. A Review of Research on Intercultural Foreign Language Teaching in China in the Last Decade. Journal of Jiaozuo Teachers College. 2019;(3):66–70. (In Chinese)

[64] Chi RB. Content Analysis of References in Domestic English Major Intercultural Communication Textbooks. Foreign Language Education in China. 2010;(2):70–4. (In Chinese)

[65] Zhang HL. Intercultural Foreign Language Teaching. Shanghai: Shanghai Foreign Language Education Press; 2007. (In Chinese)

[66] Menard- Warwick J. The Cultural and Intercultural Identities of Transnational English Teachers: Two Case Studies from the Americas. TESOL Quarterly. 2008;42: 617–640. 10.1002/j.1545-7249.2008.tb00151.x

[67] Jackson J. Cultivating cosmopolitan, intercultural citizenship through critical reflection and international, experiential learning. Language and Intercultural Communication. 2011;11: 80–96. 10.1080/14708477.2011.556737

[68] Fox RK, Diaz‐Greenberg R. Culture, multiculturalism, and foreign/world Language Standards in U.S. Teacher Preparation programs: toward a Discourse of Dissonance. European Journal of Teacher Education. 2006;29: 401–422. 10.1080/02619760600795270

[69] DeJaeghere JG, Cao Y. Developing U.S. Teachers’ Intercultural competence: Does Professional Development matter? International Journal of Intercultural Relations. 2009;33: 437–447. 10.1016/j.ijintrel.2009.06.004

[70] Parmenter LK. Becoming International in a Japanese Junior High School: an Ethnographic Study. Thesis. 1997. Available: https://ethos.bl.uk/OrderDetails.do?did=1&uin=uk.bl.ethos.361918

[71] Risager K. Language and culture pedagogy: from a national to a transnational paradigm. Clevedon: Multilingual Matters; 2007.

[72] Sun YZ, Bennett J. Toward Intercultural Education: a Dialogue between Professor Sun Youzhong and Dr. Janet Bennett. Foreign Languages and Their Teaching. 2017;293: 1–8, 146. (In Chinese)

